# Stroke injury, cognitive impairment and vascular dementia^☆^

**DOI:** 10.1016/j.bbadis.2016.01.015

**Published:** 2016-05

**Authors:** Raj N. Kalaria, Rufus Akinyemi, Masafumi Ihara

**Affiliations:** Institute of Neuroscience, Newcastle University, Campus for Ageing & Vitality, Newcastle upon Tyne, NE4 5PL, United Kingdom; Neuroscience and Ageing Research Unit, Institute for Advanced Medical Research and Training, College of Medicine, University of Ibadan, Nigeria; Department of Stroke and Cerebrovascular Diseases, National Cerebral and Cardiovascular Center, 5-7-1 Fujishiro-dai, Suita, Osaka 565-8565, Japan

**Keywords:** Alzheimer's disease, Cognitive impairment, Dementia, Microinfarcts, Neuroimaging, Post-stroke dementia, Stroke, White matter, Vascular dementia

## Abstract

The global burden of ischaemic strokes is almost 4-fold greater than haemorrhagic strokes. Current evidence suggests that 25–30% of ischaemic stroke survivors develop immediate or delayed vascular cognitive impairment (VCI) or vascular dementia (VaD). Dementia after stroke injury may encompass all types of cognitive disorders. States of cognitive dysfunction before the index stroke are described under the umbrella of pre-stroke dementia, which may entail vascular changes as well as insidious neurodegenerative processes. Risk factors for cognitive impairment and dementia after stroke are multifactorial including older age, family history, genetic variants, low educational status, vascular comorbidities, prior transient ischaemic attack or recurrent stroke and depressive illness. Neuroimaging determinants of dementia after stroke comprise silent brain infarcts, white matter changes, lacunar infarcts and medial temporal lobe atrophy. Until recently, the neuropathology of dementia after stroke was poorly defined. Most of post-stroke dementia is consistent with VaD involving multiple substrates. Microinfarction, microvascular changes related to blood–brain barrier damage, focal neuronal atrophy and low burden of co-existing neurodegenerative pathology appear key substrates of dementia after stroke injury. The elucidation of mechanisms of dementia after stroke injury will enable establishment of effective strategy for symptomatic relief and prevention. Controlling vascular disease risk factors is essential to reduce the burden of cognitive dysfunction after stroke. This article is part of a Special Issue entitled: Vascular Contributions to Cognitive Impairment and Dementia edited by M. Paul Murphy, Roderick A. Corriveau and Donna M. Wilcock.

## Highlights

• Ischaemic injury is common among long-term stroke survivors
• About 25% stroke survivors develop dementia with a much greater proportion developing cognitive impairment
• Risk factors of dementia after stroke include older age, vascular comorbidities, prior stroke and pre-stroke impairment
• Current imaging and pathological studies suggest 70% of dementia after stroke is vascular dementia
• Severe white matter changes and medial temporal lobe atrophy as sequelae after ischaemic injury are substrates of dementia
• Controlling vascular risk factors and prevention strategies related to lifestyle factors would reduce dementia after stroke

## 1 Introduction

Stroke as the third leading cause of death is considered an important cause of long-term disability and cognitive impairment. This demands enormous resources from healthcare systems [1]. The incidence of stroke varies greatly according to the age structure of the population under study. The age-adjusted annual incidence of all first-time strokes in different countries has changed considerably over the last four decades (1970–2010) [2], [3] Stroke incidence in the UK has decreased and survival after stroke has improved in the past 10 years. [4] Improved drug treatment in primary care is likely to be a major contributor to this, with better control of risk factors both before and after incident stroke. The increasing incidence of stroke in low to middle income countries over the past four decades is likely explained by health and demographic transitions in these countries [5]. However, the global burden of stroke is likely underestimated by not accounting for silent strokes, transient ischaemic attacks in many cases, vascular dementia (VaD) and long term stroke related disability in case definition.

Deaths from stroke have declined in high-income countries and many middle- and low-income countries. The key element in this decline is reduced incidence of stroke [2] but the case fatality rates have also decreased due to either lesser stroke severity or improved management [6]. Although age-standardised rates of stroke mortality have decreased worldwide in the past two decades, the absolute number of people who have a stroke every year, stroke survivors, related deaths, and the overall global burden of stroke (DALYs lost) are increasing with most of the burden in low-income and middle-income countries. The most striking increases in the number of stroke survivors (113%), DALYs lost (31%), and stroke-related deaths (36%) were in people aged 75 years and older. Increasing age is the strongest risk factor for stroke throughout the lifespan. The steep increases in incidence with age occur in both men and women. While the risk for a child under 15 years of age is 1 in 100 000, it is 1 in 33 for people aged 85 years and over. Stroke incidence more than doubles every decade after the age of 55 years until 84 years and beyond [7]. In the Oxford vascular study, stroke rates increased from 1.8/1000 individuals per year for 55–64 year olds to 17 for those aged 85 or older [8]. High blood pressure is the most treatable risk factor for both ischaemic and haemorrhagic strokes. It presents a golden opportunity for prevention and reducing the burden of stroke and post-stroke cognitive impairment.

## 2 Stroke types contributing to impairment

The clinical diagnosis of stroke is usually accurate but the precise type of stroke and exact localization may be less straightforward. Determination of the pathological type of stroke is best achieved by early brain imaging usually computed tomography (CT) or by autopsy confirmation. In western countries, cerebral infarction accounts for approximately 80% of first-time strokes, and parenchymal brain haemorrhage for 20%. In Africa, however, the burden of haemorrhagic strokes are reported to be significantly greater by more than 10% at a ratio of 66:34 ischaemic to haemorrhagic [9]. According to the recent updated definitions of the Stroke Council of the American Heart Association/American Stroke Association [10], the relative frequencies and subtypes of ischaemic and haemorrhagic infarctions may be (1) atherothrombotic, due to either (1 A) large artery thrombosis or (1B) artery-to-artery embolism; (2) cardioembolic; and (3) lacunar. The limitations of even the most advanced imaging techniques can be recognised by the inclusion of (4) infarcts of undetermined cause [11]. In infarcts of known cause, the lumen of intracranial large to medium-sized arteries is most commonly occluded by an embolus [10]. The frequency of locally formed thrombi in these arteries proved to be much lower than had been estimated previously. In contrast, a local process most often occludes small intraparenchymal penetrating arteries: thrombosis of a diseased small artery, microatheroma or microemboli from an atherosclerotic plaque occluding the origin of a penetrating artery [12]. The presence of microemboli in retinal arteries provides indirect evidence that microemboli may also enter small-calibre intracerebral penetrating arteries. However, more recent recommendations [13], [14] highlight that besides distinguishing the main aetiological categories including atherothrombotic, cardioembolic, small vessel disease and other rarer causes, aetiological classification of stroke should reflect the most likely cause without neglecting other vascular conditions that may co-exist. For example, small vessel disease often occurs in the presence of severe large vessel obstruction [12], [15].

## 3 Stroke and dementia: definitions and criteria

Dementia developing after stroke is thought to be a clinical entity to define any type of dementia occurring subsequent to stroke injury irrespective whether it involves vascular, neurodegenerative or mixed processes. It can therefore entail a complex aetiology with varying combinations of large and small vessel disease as well as non-vascular neurodegenerative pathology. The development of dementia after stroke depends on several factors including the location and volume of the stroke, degree of related neuronal damage, presence of pre-existing cognitive impairment or other cerebral pathology. The direct influence of any specific genetic factor(s) is not clear. However, the heritability estimate for all ischaemic strokes was determined to be 38% but varied considerably by subtype with the greatest associated with large vessel (40%) and cardioembolic disease (33%) and lowest with small vessel disease (16%) [16].

Cognitive impairment or dementia after stroke is predominantly defined by dementia that occurred within three months after stroke onset. Irrespective, many stroke survivors develop delayed dementia beyond three months or only after recurrent stroke(s). The recognition of cognitive impairment in the acute phase after stroke may offer vital information to the clinician for early cognitive rehabilitation [17] and preventing early fatality by improved management [18].

**Fig. 1:**
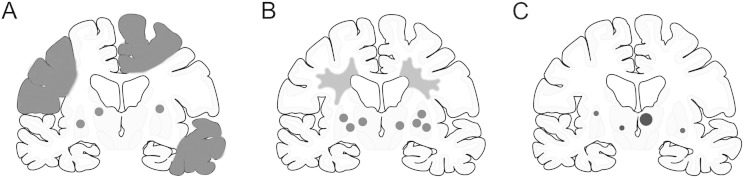
Localization of infarcts or tissue changes associated with development of dementia after stroke. Dementia associated with different cerebrovascular pathologies. Subtype I may result from large vessel occlusion (atherothromboembolism), artery-to-artery embolism or cardioembolism. Subtype II usually involves descriptions of arteriolosclerosis, lipohyalinosis, hypertensive, arteriosclerotic, amyloid or collagen angiopathy. Subtype III is caused by infarcts in the ‘strategic’ areas such as the thalamus and hippocampus and may involve several risk factors including cardioembolism and intracranial small vessel disease. Shaded areas in each outline coronal show locations of lesions. Small dots depict small infarcts and microinfarcts, although the size of the latter can only be appreciated from microscopical images. Currently reported studies (and unpublished data) show that the estimated % cases for the three subtypes are as follows: Subtype I, 20–40%; subtype II, 40–50% and subtype III, 10–15%. The risk factors associated with particularly Subtype I can be varied including hypertension, carotid artery disease or atherosclerosis, cardio embolism (mostly atrial fibrillation) and coronary artery disease. Subtype II may involve hypertension, diabetes mellitus, hyperlipidaemia, hyperhomocysteinaemia, chronic kidney disease, infection and obstructive sleep aponea. Lifestyle factors such as smoking, obesity and alcohol abuse are other factors. These subtypes would include dementia among post-stroke survivors who fulfil the National Institute of Neurological Disorders and Stroke and Association Internationale pour la Recherché et l'Enseignement en Neurosciences (NINDS-AIREN) criteria for probable vascular dementia.

**Fig. 2:**
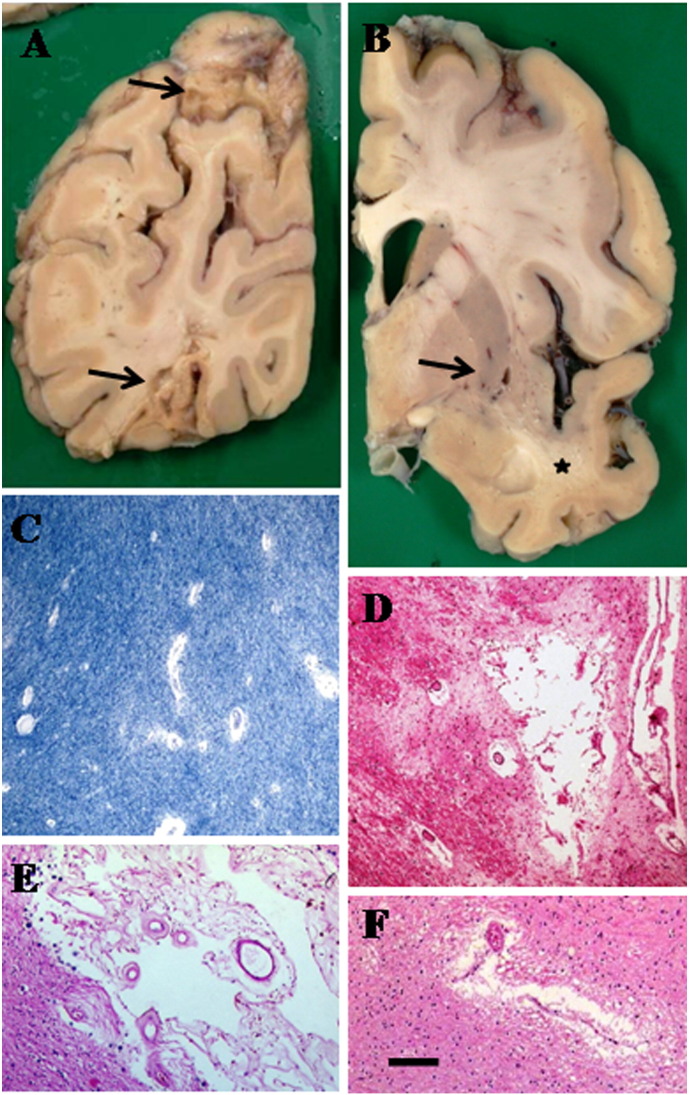
Pathological changes found in stroke Subtypes I to III involving large artery and small vessel diseases in elderly stroke survivors. A, Large infarcts (arrows) in the parietal lobe of 80-year old woman with cognitive impairment. This fits the classification of Subtype I in Fig. 1. B: Lacunes (arrow) and WM lesions in external capsule in the basal ganglia of a 78-year-old man with cognitive impairment. Note also WM rarefaction in the temporal limb (star symbol). C: Perivascular spaces (dilatation) and demyelination in WM (Luxol fast blue stain). D, Microinfarct in the caudate with some perivascular dilatation (HE, Haematoxylin and Eosin). E, Small infarct in the temporal pole (HE). C-E are typical small lesions in subtype II. F, A microinfarct in the thalamus. Moderate gliosis in the surrounding region is also evident. Thalamic infarcts can occur as 0.5 cm lacunes in the coronal section in B. Magnification Bar: A and B = 2 cm; C, D and F = 50 μm, E = 100 μm.

Recent prospective studies suggest stroke survivors may unmask or trigger varied pathologies including those attributed to subcortical VaD, multi-infarct dementia and even strategic infarct dementia [19], [20], [21] (Fig. 1). Given this definition, most cases of dementia after stroke may be described under the umbrella term of vascular cognitive impairment (VCI) [22], [23], which is introduced to incorporate the full spectrum of cognitive changes related to all causes of vascular disease from VCI no-dementia to frank dementia of vascular origin. It is suggested that dysfunction of the neurovascular unit and mechanisms regulating cerebral blood flow particularly in the deep white matter (WM) are important components of the pathophysiological processes underlying VCI. The continuum of VCI is also discussed broadly under the rubric of vascular cognitive disorders (VCDs) [24], which comprise many diseases, each with varying severity and patterns of dysfunction. The categorical diagnosis of VCDs encompasses mild impairment, pre-dementia, and dementia syndrome, and major VCD category is equivalent to dementia as adopted in the DSM-5 criteria. Overall, dementia after stroke fits the categorization of severe VCD [24]. (See Fig. 2.)

The cognitive domains involved in the development of dementia after stroke may also vary depending on stroke characteristics such as stroke type, volume, numbers, location and severity. Regarding the stroke type, patients with ischemic strokes usually have higher survival rates than do those with hemorrhagic strokes, which explains why ischemic strokes lead to psychiatric morbidity more frequently than do hemorrhagic strokes [25]. Important critical locations also include dominant hemisphere and lesions affecting the prefrontal–subcortical circuit that mediates executive dysfunction [26], [27]. Frontal lobe functions comprising processing speed, reaction time, working memory and executive task measures are most affected [28]. A single large cortico-subcortical brain ischemic lesion, if located in an area that is functionally critical for cognition, may present with acute cognitive deterioration. Strategic infarct dementia is attributed to locations in the angular gyrus, the medial frontal lobe, and the inferomedial portion of the temporal lobe, all of which may be caused by large-vessel pathology [29]. Bilateral hippocampal or thalamic infarctions and unilateral thalamic infarctions are other examples of strategically localised infarctions that are reported to cause dementia (Fig. 1, subtype III). Strategic infarction dementia may be caused by damage to the components of Papez (hippocampal memory loop) [30] or Yokovlev circuits. However, dementia resulting from strategic infarctions may not take into account the influence from other lesions [31].

In post-stroke cohorts, the presence of executive syndrome and depression is the predictor of poor long-term survival rather than depression itself [32]. Dementia and depression also interact with each other in the post-stroke period [33]. If there is preceding dementia, stroke worsens the cognitive impairment. This is called pre-stroke dementia with possibility of co-existing neurodegenerative pathology as a cause of dementia [34]. Progressive dementia without any symptomatic stroke but with only radiologically proven cerebrovascular diseases may not be diagnosed with dementia after stroke because cerebrovascular lesions such as WM changes and apparently silent lacunar infarcts are common in demented elderly with mixed pathology consisting of Alzheimer's disease (AD) and cerebrovascular disease.

Dementia associated with cerebrovascular diseases is clinically diagnosed using the widely accepted DSM IIIR or IV criteria. The apparent “gold standard” for diagnosis has been based on the results of the extensive neuropsychological examination, clinical presentation, and information from a close relative as well as the usefulness of R-CAMCOG [35] or the Montreal Cognitive Assessment (MoCA) [36], [37], [38]. Such short screening tests although are useful for both clinical and research purposes, their sensitivity is limited and there is no clear consensus as to which test is the most appropriate so some have used both or devised wide-ranging test batteries [27], [34], [39].

Dementia after stroke may incorporate different types of dementias but these there is hardly any pathological confirmation for most of the clinical or prospective studies [21], [40] even where there is strong contribution of neurodegenerative disease. The Newcastle CogFAST study [21] incorporating pathological examination showed that almost 75% of demented stroke survivors fit current criteria for VaD [22], [29], [41], whereas the rest had mixed pathology with either Alzheimer type of lesions or Lewy bodies. Consistent with these observations, Mok and colleagues [42] have elegantly shown that 70% of the stroke survivors do not retain AD-like Pittsburgh compound B binding suggesting classical neurodegenerative pathology is not a major contributor to cognitive impairment after stroke injury. Thus, majority of stroke survivors with an appropriate clinical diagnosis of dementia and neuropsychometric assessment develop VaD. It would be reasonable to assume that many of these subjects will have small vessel disease type of pathology (Fig. 1, subtype II and Fig. 2) although there may be some burden of small cortical infarcts and microinfarcts [21], [40].

## 4 Frequencies of impairment after stroke

As the risk of death from strokes has declined, the number of stroke survivors with cerebral compromise and cognitive dysfunction has increased. Stroke survivors are at increased risk of cognitive impairment [21], [43]. Meta-analysis of data from several studies yielded estimates of 1-in-10 patients being demented prior to a first stroke, 1-in-10 developing new dementia soon after a first stroke, and over 1-in-3 being demented after a recurrent stroke [44], [45]. The development of cognitive impairment and incident dementia after stroke appears relatively common following stroke [19], [45]. While not to be confused with periods of delirium as an immediate consequence after stroke injury [46], [47], cognitive decline following an index stroke could be insidious with the latent appearance of dementia especially in patients with small cortical and subcortical infarcts.

Cognitive impairment after stroke is a frequent but neglected consequence compared to other neurological deficits such as sensory or motor impairment [48]. However, not all strokes result in cognitive impairment but stroke significantly increases the risk of dementia [49]. In community-based studies with adjustment for age, the prevalence of dementia in people with a history of stroke is ~ 30% with 3·5–5·8 times higher than in those who did not have any detectable stroke injury [50]. The incidence of dementia in older people with a longer follow-up time increases from 10% at 1 year to 32% after 5 years [19]. The pooled prevalence estimates of dementia after stroke less than one year after the stroke ranged from 7.4% in population-based studies of first-ever stroke, in which pre-stroke dementia was excluded, to 41.3% (29.6–53.1) in hospital-based studies of recurrent stroke, where pre-stroke dementia was included. The cumulative incidence of dementia after the first year was slightly greater (3.0%, 1.3–4.7) per year in hospital-based studies than expected on the basis of recurrent stroke alone [45]. In the Newcastle CogFAST (cognitive function after stroke) study, we reported that although 41% were stable and 50% improved in cognition at 15 months, survivors who were dementia free at three months after the stroke [51], a substantial proportion of these subsequently progress to delayed dementia [21]. In the parallel prospective CogFAST- Nigeria study, 40% of older stroke survivors had cognitive impairment without dementia and 8.4% were demented three months after stroke [39]. Consistent with other studies, utilising the three month design [52], [53], [54] before follow up, delayed dementia after stroke was estimated to occur in at least 25% of the subjects with various risk factors. In addition, the Helsinki study [55] has most recently reported that up to 83% of stroke survivors show impairment in at least one whereas 50% are impaired in multiple (> 3) cognitive domains. Importantly, 71% of cases with good clinical recovery at 3 months still exhibited cognitive impairment in memory, visuoconstructional or executive functions.

## 5 Risk factors for cognitive impairment after stroke

In tandem with the age-related increased incidence of stroke itself, older age is the strongest risk factor for VCI and dementia after stroke (Table 1). This is consistent with the high incidence of stroke or cerebrovascular related dementia in the elderly [7], [21]. In the CogFAST studies [21], [39], we found that the mean age of cognitively impaired subjects was greater with expectedly lower CAMCOG scores than the non-demented stroke subjects.

Hypertension is the most common modifiable risk factor for stroke. Elevations in both systolic and diastolic blood pressures are associated with increased risk of stroke and by extension stroke related dementia (Table 2). In addition to severity of increased blood pressure, the duration of hypertensive state would be an important determinant of dementia after stroke. When diabetes is present, hypertensives are even at a higher risk of stroke and cognitive impairment [7]. Other risk factors for dementia after stroke include recurrent stroke, pre-stroke dependency, pre-stroke cognitive impairment, development of apathy or quality of life with lower educational status and history of diabetes mellitus [19], [39], [42], [45], [53], [56], [57]. Post stroke cognitive impairment is then not surprisingly associated with poor functional outcomes.

Studies from general community populations indicate that VaD is more frequent in males than in females, but most studies suggested no substantial gender difference for the risk of dementia after stroke. Recent findings suggest this is attributable to neuroprotective role of adiponectin because of the relatively steeper decline of serum adiponectin levels associated with ageing in females than in males [58]. Epileptic seizures, sepsis, cardiac arrhythmias and congestive heart failure are listed as other risk factors of incident dementia after stroke [59]. It is not surprising that atrial fibrillation and nephropathy independently contribute to the risk in addition to older age, previous mental decline, and stroke severity [52].

The risk of dementia is likely more when vascular comorbid conditions occur. Thus, hypertension, atrial fibrillation, diabetes mellitus, myocardial infarction, and congestive heart failure can often co-exist in various proportions [19], [42], [59], [60]. Consistent with this, our prospective longitudinal CogFAST study [21] showed that the presence of 3 or more cardiovascular risk factors increased risk of dementia or death by 4-fold in the elderly stroke survivors. These observations contrast with a systematic review [61], where the findings concluded that the effect of stroke on dementia incidence in the population was explained by recurrent stroke rather than cardiovascular risk factors. A number of studies has found significant effects of individual vascular risk factors in both early and delayed dementia after stroke but have not examined their cumulative effect. However, metabolic syndrome, a clustering of several cardiovascular risk factors may well affect dementia after stroke through ‘metabolic–cognitive syndrome’ [62]. Another longitudinal study [63] showed that patients with dementia after stroke had a higher prevalence of several vascular risk factors including hypertension, diabetes, atrial fibrillation, previous myocardial infarction and history of transient ischaemic attack (Table 2). However, these associations were not found in other studies with shorter follow up [52], [53], [64], [65], [66]. Independent contribution of vascular risk factors or disorders to the development of dementia following stroke still needs consensus [67]. Since the rates of dementia after stroke may continue to rise in a relatively linear fashion [45], they illustrate that stroke, vascular risk factors, or co-existing neurodegenerative changes make the brain more vulnerable to dementia in the longer term. The role of genetic influences in cognitive impairment or dementia per se after stroke or VaD is not clear. So far only one genome-wide association study involving 67 subjects, who developed incident VaD over a mean follow-up of 9.3 years has identified a single variant on the X chromosome near the androgen receptor gene [68]. This locus has not been linked to other stroke manifestations and the association with VaD, however, still remains to be independently confirmed. Previous results reporting on the significance of specific candidate genes such as angiotensin converting enzyme gene [69], alpha-1-antichymotrypsin [69], [70] or apolipoprotein E (*APOE*) gene [69], [71], [72] as risk factors for dementia after stroke are not entirely in agreement. In older stroke patients with early cognitive impairment, the presence of an *APOE* ԑ4 allele was reported to be associated with greater progression of cognitive decline [73]. However, as heritability of stroke may also increase risk of impairment, it is noteworthy that several candidate genes were recently identified to be associated with large artery and cardioembolic disease (Table 3). The majority of genetic variants identified has been associated with specific subtypes of ischaemic stroke suggesting that the subtypes bear distinct genetic compositions and pathophysiological mechanisms [74]. To date the association of single nucleotide polymorphisms (SNPs) in only 5 loci seems to be most consistently replicated with the most robust significance (Table 3).

Variants in genes associated with familial small vessel diseases of the brain [75] are also associated with cognitive impairment after ischaemic or haemorrhagic injury (Table 3). Among these most recent developments include the *NOTCH3* and *HTRA1* genes linked to cerebral autosomal dominant (CADASIL) and recessive (CARASIL) disorders. Both common and rare SNPs in the NOTCH3 gene show increased risk of age-related WM changes in hypertensive subjects [76]. Similarly, seven variants of the *HTRA1* gene were identified to be associated with hereditary SVD of unknown aetiology [77]. Recently, three intronic SNPs in the *COL4A2* gene were identified to be associated with symptomatic small vessel disease, particularly WM disease and deep intracerebral haemorrhage [78],

## 6 Neuroimaging determinants in relation to neuropathology

Neuroimaging has been very useful to recognise that a combination or interaction of different types of brain lesion, including neurodegenerative markers, and preexisting underlying processing are players in the development of cognitive decline after clinical stroke. Brain lesion correlates of dementia after stroke include a combination of infarct features (volume, site), the presence of WM changes (extent, location), as well as brain atrophy [79]. However, strategic site lesion, baseline stroke severity, greater severity of WM changes and medial temporal lobe atrophy appear the strongest contributors of subsequent impairment [42], [80], [81], [82], [83], [84]. In the 24 year Dijon study [63], the prevalence of dementia after stroke associated with lacunar strokes was seven times higher than other types of stroke including intracerebral haemorrhage. Pre-stroke medial temporal lobe atrophy [85], [86] or silent brain infarcts and microbleeds, and extensive WM changes were associated with an increased risk of post-stroke memory dysfunction, a prerequisite for the diagnosis of dementia after stroke. Severe WM changes associated with subcortical stroke injury in turn may influence cortical grey matter [87], [88] to contribute to impairment. The presence of other pathologies including AD-like changes is another factor that worsens cognition and increase incident dementia after stroke or transient ischaemic attack [34].

## 7 Mechanisms of cell death leading to dementia

Much of the current knowledge of the ischaemic injury cascade comes from experimental studies. The cascade consists of a complex series of events which are highly heterogeneous [89] and evolve over minutes to days and weeks after the initial hypoperfusive event. The main stages include energy failure due to disruption of blood flow, excitoxicity, calcium overloading, oxidative stress, blood- brain barrier (BBB) dysfunction, microvascular injury, haemostatic activation, injury - related inflammation and immune responses, and cell death involving neurons, glia and endothelial cells. Microvascular damage and disruption of the BBB, which may occur days later, lead to vasogenic oedema and can also cause haemorrhages. At the same time, the tissue may undergo complex range of reparative and remodelling responses including angiogenesis to limit damage and improve outcome. These events are curtailed in ageing brains such that the parenchyma is irreparably damaged which then contributes to cognitive dysfunction.

Experimental studies suggest neuronal death following ischaemic injury is largely attributed to necrosis. However, recent developments indicate that significant cell death occurs by apoptotic as well as hybrid mechanisms (e.g. necroptosis) along an apoptosis–necrosis continuum. While the infarcted core is necrotic be it macro- or microinfarction, within the penumbra caspase-mediated apoptosis is activated although secondary necrosis results from failure to implement the apoptotic signalling pathways fully, as they require energy. Ischaemic injury triggers two general pathways of apoptosis. The intrinsic pathway originates with mitochondrial release of cytochrome *c* and subsequent stimulation of caspase-3 whereas the extrinsic pathway is initiated by the activation of cell surface death receptors, which belong to the tumour necrosis factor superfamily, by Fas ligand, resulting in the stimulation of caspase-8. There is some evidence that ischaemic cell death may also be mediated by autophagy, which is activated during cerebral ischaemia for the bulk removal of damaged neuronal organelles and proteins [90], [91]. Molecular markers of the autophagic process during ischaemic injury include increased production of microtubule-associated protein 1 light chain 3 (LC3-II), Beclin-1, lysosome-associated membrane protein 2 and lysosomal cathepsin B. Oxidative and endoplasmic stresses in cerebral ischaemia are proposed to be potent stimuli for autophagy in neurons [92]. There is also cross-talk between pathways involved in autophagy and apoptosis and both processes occur in parallel, as suggested by upregulation of both autophagic and apoptotic markers in the ischaemic penumbra [90]. It is not unlikely that autophagy after ischaemic injury [93] is a contributor to parenchymal changes leading to impairment.

Neuroinflammation and immunodepression are also associated with stroke, ageing, and infection. These likely have a detrimental role in cognitive function after stroke [94], [95], [96], [97]. Diminished or impaired inflammatory mechanisms are likely another factor in the pathways leading to dementia. Thus, besides immunosenescence, cerebral atrophy is another factor why reactive cells including microglia and astrocytes exhibit a dampened cytokine response in the production of IL-6 and IL-8 in those stroke subjects that develop dementia compared to those who do not. In contrast, C reactive protein was not altered between dementia and non-dementia subjects. Strategies that enhance anti-inflammatory cytokines and boost the neuroimmune system may be beneficial for preventing cognitive dysfunction, especially after stroke [98].

Compared to neuroimaging correlates the pathological substrates associated with dementia after stroke or VaD have generally lagged behind. This is particularly true in defining those substrates associated with executive dysfunction. There is selective atrophy (30–40%) of pyramidal cells in layers III and V of the dorsolateral prefrontal cortex compared to the anterior cingulate and orbitofrontal cortices of the frontal lobe in subjects with dementia after stroke, VaD and, of mixed and AD versus normal ageing controls and those who did not have dementia after stroke [99]. These findings indicated neuronal atrophy occurs irrespective of the presence of amyloid or neurofibrillary or tau pathology. The neuronal volume changes were also related to cognitive dysfunction shown by lower total revised Cambridge Cognition Examination (CAMCOG), orientation and memory scores and clinical dementia ratings. In further morphometric analysis of the dorsolateral prefrontal cortex, it was shown that neither diffuse cerebral atrophy nor neocortical thickness explained the selective neuronal volume effects. However, neurofilament protein SMI31 immunoreactivity was increased in subjects with dementia after stroke and VaD compared to non-demented stroke subjects and correlated with decreased neuronal volumes in both dementia after stroke and VaD subjects [99]. These findings possibly relate to causal relationship between incident subcortical infarcts or acute infarcts and morphological alterations in connected cortical regions, which exhibit cortical thinning or atrophy [87], [100]. Conceivably, secondary neurodegeneration within the cortical grey matter may occur after myelin loss and axonal damage in the WM.

## 8 Pathophysiology of the WM and the blood brain barrier

Constant perfusion of the WM by deep penetrating arterioles is essential for functioning of axons and oligodendrocytes. Depending on the duration and severity of ischaemic or oligaemic injury, several features may be readily evident in the WM. These include activated microglia, clasmatodendritic astrocytosis, myelin breakdown, presence of axonal bulbs and degeneration, reactivation and loss of oligodendroglia. During subsequent (chronic) periods of arteriolar changes, perivascular spacing and apoptotic oligodendroglia are seen accompanied by degeneration of myelin and expression of hypoxia markers. [101].

WM hyperintensities as seen on brain T2-weighted or FLAIR magnetic resonance imaging (MRI) are associated with varying degrees of cognitive dysfunction in stroke, cerebral small vessel disease and dementia. The pathophysiological mechanisms within the WM accounting for cognitive dysfunction remain unclear. Stroke patients with more severe WM changes have an increased risk of recurrent strokes. Thus, the presence and severity of WM changes seen on MRI may be predictive of post-stroke dementia [19], [82], [102], [103]. However, WM hyperintensities may not be predictive of subsequent decline in all stroke survivors and clearly other anatomical substrates appear more involved [85], [102]. Ischemic WM changes are most prominent in the frontal lobe [104] and appear linked with frontal -subcortical disconnection [27], [105]. The WM changes consist of myelin rarefaction with shrunken oligodendrocytes and axonal abnormalities resulting from vascular insufficiency and a chronic hypoxic state [106], [107], [108]. Independent effects of WM changes in dementia among stroke patients needs to be verified by simultaneously taking into account all other MRI findings as other types of brain lesions, such as cerebral atrophy and silent infarcts are strongly correlated with WM changes. Microstructural changes of normal appearing WM as evident in diffusion tensor imaging may be a better predictor of cognitive decline in patients with WM hyperintensities [109]. It also apparent that the edge of WM hyperintensities is a predilection site for lacunes, most of which typically occur proximal to pre-existing WM hyperintensities with regard to the anatomical course of perforating vessels. These observations highlight the concept of the WM hyperintensity penumbra [110].

Recent clinicopathological studies [111] showed that post-stroke survivors who had exhibited greater frontal WM hyperintensities volumes which predicted shorter time to dementia onset, consistently exhibited disruption of gliovascular interactions and BBB damage. In contrast to normal appearing glial fibrillary acid protein immunopositive (GFAP +) astrocytes, the percentage of clasmatodendrocytes was increased by > 2-fold in demented compared to non-demented subjects and by 11-fold in older normal controls versus young controls in the frontal WM. These were associated with aberrant co-localization of aquaporin-4 in retracted GFAP + astrocytes with disrupted end-feet juxtaposed to microvessels. High ratios of clasmatodendrocytes to total astrocytes in the frontal WM were consistent with lower Mini-Mental State Examination and the revised CAMCOG scores in demented subjects. Thus, clasmatodendrosis appears another pathological substrate, linked to WM hyperintensities and frontal WM changes, which contributes to delayed dementia after stroke injury [111].

## 9 Medial temporal lobe and global atrophy

Medial temporal lobe and global atrophy are both shown to be associated with dementia after stroke [19] (Table 1). If medial temporal lobe atrophy is considered a marker of AD, the development of dementia after stroke in subjects with medial temporal lobe atrophy may be caused by the concomitant neurodegenerative process that was ongoing in the preclinical phase at the time of stroke occurrence. Previous studies found that in elderly stroke survivors, medial temporal lobe atrophy was associated with shorter time to dementia [86] but it was more strongly associated with subsequent cognitive decline than were WM changes [85], which suggested a greater role for Alzheimer-type pathology than cerebrovascular lesions in the development of delayed cognitive impairment after the onset of clinical stroke. This was further supported by the findings that reductions in blood flow, assessed by arterial spin labelling, in the posterior artery territories and in volumes of the hippocampal formation were similar in dementia after stroke and AD subjects [65], [112]. In accordance with these findings, another study showed that increasing severity of medial temporal lobe atrophy was associated with amnestic VCI no dementia (OR = 2.69; 95% CI = 1.21–5.99) and amnestic MCI (OR = 5.20; 95% CI = 2.41–11.23) compared to non-amnestic VCI no dementia in post stroke survivors [113]. Moreover, the impaired post stroke episodic memory function may be caused by reduced medial temporal lobe functionality [114]. Significant correlation between WM changes and medial temporal lobe atrophy was similarly found in a cohort of African stroke survivors [57]. However, WM changes in the frontal and parieto-occipital regions correlate with hippocampal atrophy, suggesting that there is a tangible link between vascular pathology and hippocampal atrophy [115]. That such a link exists, there is selective hippocampal neuronal shrinkage which does not only appear to be an important substrate for AD but also delayed dementia after stroke in the absence of any neurodegenerative pathology [116]. This is consistent with the findings in animal models that long-term hypoperfusion does not require co-existing neurodegenerative changes to induce hippocampal atrophy [117] and demonstrates the vascular basis for hippocampal neurodegeneration and dementia.

## 10 Silent brain infarcts, microbleeds and cerebral amyloid angiopathy

Several studies have consistently reported that cerebral silent infarcts detectable with computed tomography or MRI were independently predictive of dementia after stroke [118], [119]. Silent infarcts may be more important to the delayed onset of dementia in patients with clinical stroke because presence of silent infarcts is associated with dementia after stroke detected in the third year, but not in the second year after the index stroke [120]. In addition, microbleeds are radiological hallmarks of cerebral amyloid angiopathy (CAA) that can be detected with the T2*-weighted gradient-echo sequence of MRI. CAA may cause cognitive impairment because of multiple substrates, which include cerebral microbleeds, microinfarcts and WM changes (leukoaraiosis). WM pathology is quite common in CAA but WM changes have not been consistently correlated with CAA [121], [122], [123]. CAA, however, is a cause of cortical microinfarcts (50–500 μm in diameter) that are invisible on conventional 1.5 and 3 Tesla MRI [124], [125], [126]. While observations on the occurrence of stroke and dementia after stroke in patients with microbleeds are not generally available [127], a recent study in a large cohort of patients in a memory clinic found a relatively high frequency of microbleeds in patients with VaD (65%), AD (18%), and mild cognitive impairment (20%) [128]. This clinic-based study is consistent with the neuropathological observations, which showed that severe CAA and cerebral cortical microinfarcts are in tandem important substrates of cognitive decline [129]. Changes in the hemodynamics such as hypotension in the presence of CAA may be indicated as a key factor in the genesis of cortical watershed microinfarcts [85], [130].

## 11 Cognitive dysfunction prior to stroke

The risk of dementia after stroke is increased in patients with pre-stroke cognitive decline, with about one-third of patients meeting the criteria for AD and two-thirds meeting the criteria for VaD [131]. This has led to the formulation of the term “pre-stroke dementia” [131]. Preexisting brain structural changes may have impact on the occurrence of dementia after stroke. These preexisting changes are likely related to different mechanisms possibly incorporating parallel processes i.e. neurodegenerative and vascular that result in the development of dementia [132]. Brain atrophy and medial–temporal lobe atrophy may underlie AD pathology, while WM changes may be more prone to subcortical VaD. However, as previously emphasised [133], there is an overlap between vascular and degenerative mechanisms responsible for the pathogenesis of VaD such that in the oldest old there is high burden of mixed pathology [107]. Only one single factor cannot explain the development of dementia related to large-vessel pathology, as the aetiology is probably multi-factorial, where stroke characteristics (stroke type, volume, number, location and severity) as well as host factors such as comorbidities contribute to the risk independently. In the majority of the cases, several mechanisms interact to exceed the critical threshold for normal cognition, and dementia subsequently occurs. Thus, even marginally increased burden of amyloid and neurofibrillary pathology above normal ageing but insufficient for pathological diagnosis of AD likely adds to the tissue degenerative process leading to dementia after stroke. This important issue could be further investigated using positron emission tomography (PET) imaging for amyloid [134] or tau [135].

## 12 Preventive strategies

Any measure that reduces or controls vascular disease would be preventative for dementia after stroke [136]. In recent years, a variety of lifestyle factors including diet and physical activity that reduce or abate vascular disease has been highlighted for prevention of dementia. These would all be equally applicable in the prevention of dementia after stroke. Despite its high prevalence, the treatment options for dementia after stroke are limited. In fact, patients with cognitive impairment or dementia subsequent to stroke injury may often be less treated with aspirin or warfarin than non-demented individuals [137]. There are no drugs so far approved for the treatment of VaD per se. Cholinesterase inhibitors and memantine may have a beneficial effect, which may be due to co-existing AD pathophysiology [138]. As most vascular risk factors and disorders are modifiable (Table 2), understanding the role of vascular risk factors in dementia is crucial for devising strategies for the treatment of dementia after stroke [139]. Nevertheless, as dementing disorders, once established, are not curable, implementation of primary treatment of vascular disorders seems to be the most promising for reducing the burden of not only dementia after stroke but also dementia in general.

Stroke is related to two- to nine fold increase in risks of dementia [43]; therefore, adequate primary prevention and neuroprotective intervention after stroke occurrence or recurrent events [140] will have enormous impact. As stated above hypertension is the most modifiable risk factor for stroke. For primary prevention, trials involving various classes of antihypertensives suggest any antihypertensive could be useful to reduce risk [141]. For secondary prevention, the combination of ACE inhibitors and diuretic agents has been recommended [142]. However, trials assessing secondary prevention usually exclude patients with dementia. Results collected between 1995 and 2011 from the community-based South London Stroke Register [139] showed that, in patients with ischemic strokes without a history of atrial fibrillation, there was a significantly reduced risk of cognitive impairment associated with the use of antihypertensives (relative risk, 0.7 for diuretics; 0.8 for angiotensin-converting enzyme inhibitors; and 0.7 for their combination) when clinically indicated. In addition, there was a tendency of reduced risk of cognitive impairment associated with the use of a combination of aspirin and dipyridamole (relative risk, 0.8) and statin (relative risk, 0.9) [139]. Protective effects against cognitive impairment were also observed in patients on the combination of antihypertensives, antithrombotic agents, and lipid-lowering drugs (relative risk, 0.55). Educational attainment has been found to protect against cognitive impairment in numerous disorders including MCI, AD and VaD [138]. Educational achievements together with occupational complexity and social engagement constitute the ‘Cognitive Lifestyle’ paradigm, which has been associated with a reduced long-term risk of dementia and found to be associated with neurotrophic changes in the prefrontal lobe consistent with a compensatory process. Physical activity and healthy diet including fish intake have also been shown to be protective against post-stroke dementia [39]. These rather modestly inexpensive measures have immense implications for public vascular and brain health especially in low resource settings.

## 13 Summary

Cognitive impairment after ischaemic stroke injury is common in different populations. Although dementia after stroke is a clinical entity, current neuroimaging and pathological studies suggest that majority of older age-related dementia after stroke can be classed as VaD. Emerging small vessel disease associated genetic traits, severe WM changes and medial temporal lobe atrophy are important features in the development of dementia after stroke injury. The well-established relationship between vascular risk factors and dementia provides a rationale for the implementation of interventions. Control of vascular disease risk and prevention of recurrent strokes are obviously key to reducing the burden of cognitive decline and dementia after stroke.

## Competing interests

The authors declare no competing interests.

## Transparency document

Transparency document

## Figures and Tables

**Table 1:** Risk factors of delayed dementia after stroke injury.

Demographic features	Odds ratios (p = 0.05–0.01)
Advanced age	6.6 for > 65 years⁎; 1.05–1.2 per year
Genetic traits	> 1.5
Low education	2.5
Stroke characteristics	
Transient ischaemic attack	1.83†
Recurrent stroke	2.3
Multiple infarcts	2.5
Strategically located infarcts	NA
Stroke severity	2.5

Neuroimaging markers of brain lesions	
Silent brain infarcts	1.8
White matter lesions	2.5
Medial temporal lobe atrophy	2.69–5.2††
Cerebral atrophy (global/temporal lobe atrophy)	2.6
Cerebral microbleeds	NA‡

Data taken from several publications: [19], [42], [45], [143], [144]. Abbreviations: NA, not available; VCI, vascular cognitive impairment.

⁎ This study showed rather high risk with age [144].

† OR in post stroke patients with pre-stroke TIA less than 4 weeks was determined to be 1.83 (95% CI 1.32-2.52).

†† ORs of post stroke amnestic VCI and MCI groups compared with non-amnestic VCI- no dementia group [113].

‡ Cerebral microbleeds predicted frontal-executive impairment with OR 8.4 up to 5.7 years follow up [145].

**Table 2:** Modifiable or treatable risk factors for dementia after stroke injury.

Risk factor	Clinical features and targets for modification
Hypertension	Both systolic and diastolic pressures increase risk; > 140/90 mm Hg
Atrial fibrillation	Both chronic and paroxysmal AF confer risk of stroke. AF involved in ~ 10% of all strokes; in > 80 year olds it is ~ 36%. Anticoagulants including aspirin suggested but they are not without risk. Not recommended for those who develop dementia.
Diabetes mellitus Type II	Risk of stroke can be independently increased by 1.8 to 6 fold. Strategies to reduce risk of stroke are focused in reducing co-morbidity with hypertension.
Dyslipidaemia	Elevated cholesterol and LDLs and lower HDLs increase stroke. Consistent reduction of stroke risk by use of statins but only marginally effective in dementia
Cardiac and Carotid arterial diseases	Arterial stenosis or occlusion caused by atherosclerotic plaques is well-known risks for stroke and cerebral hypoperfusion. Both asymptomatic and symptomatic arterial disease are associated with cognitive impairment.
High homocysteine	Elevated homocysteine (> 13 mg/ml) is considered risk for vascular disease related cognitive impairment but not widely accepted. Diet folate supplementation can lower homocysteine.
Obesity	BMI of > 25 and increased abdominal fat stroke predictors of stroke risk. Body weight reduction reduces risk of stroke.
Metabolic syndrome	Cluster of modifiable subclinical and clinical conditions including glucose tolerance, elevated blood pressure, low HDL and abdominal obesity increases risk of stroke. Aggressive strategy to reduce multiple components would reduce risk.

Risk factors mostly associated with ischaemic stroke rather than dementia after stroke. Data derived from several references: [141], [142], [146], [147], [148], [149], [150]. Among others chronic kidney disease as a marker of vascular risk factors such as hypertension and diabetes is associated with small vessel disease and cognitive impairment [151]. Cigarette smoking and excessive alcohol consumption may also contribute to impairment by increasing stroke risk. Abbreviations: AF, atrial fibrillation; BMI, body mass index; HDL, high-density lipoprotein; LDL, low-density lipoprotein.

**Table 3:** Common variants of widely confirmed genes associated with sporadic stroke phenotypes⁎.

Stroke Type/vascular disorder	Candidate gene (or near locus)	Chromosome locus	Gene variants††	Protein type/function	Predicted dysfunction(s) or pathology
CADASIL†	*NOTCH3*	19p13.2-p13.1	Non-synonymous SNPs (H170R, P496L, V1183M, L1518M, D1823N and V1952M)	Transmembrane cell signalling receptor	Aberrant cell–cell signalling, activates unfolded protein response and impaired gene transcription (NICD)
CARASIL†	*HTRA1*	10q25.3-q26.2	Heterozygous variant R166L	A serine protease	Promote serine-protease-mediated cell death, suppress TGF-beta expression
Small vessel disease (with ICH)†	*COL4A2*		rs9521732; rs9521733; rs9515199 (intronic SNPs)	Collagen IV	Basement membrane proteins associated with complex structure. COL4-related angiopathies are caused by mutations in *COL4A1*/*COL4A2*
Large artery atherosclerosis	*HDAC9*	7p21.1	rs2107595	Unknown	Function associated with large artery atherosclerosis
Large artery atherosclerosis	*TSPAN2*	1p13.2	G allele at rs12122341	Unknown	Gene close to member of transmembrane 4 (tetraspanin) superfamily of proteins, which mediate signal transduction to regulate cell development, activation, growth, and motility. Associated with large artery atherosclerosis-related stroke
Large artery atherosclerosis	*SUPT3H*/*CDC5L*	6p21.1	rs556621	Unknown	Function associated with large artery atherosclerosis-related stroke
Cardioembolism	*ZFHX3*	16q22.3	rs879324	Unknown	Function associated with AF, which common in elderly
Cardioembolism	*PITX2*	4q25	rs6843082	Unknown	Function associated with AF, which common in elderly
All strokes (small arteries)	*ALDH2*	12q24.12	rs10744777	Unknown	Function varied as gene associated with all ischemic stroke subtypes

Abbreviations: AF, atrial fibrillation; BM, basement membrane; CADASIL, cerebral autosomal dominant arteriopathy with subcortical infarcts and leukoencephalopathy [159]; CARASIL, cerebral autosomal recessive arteriopathy with subcortical infarcts and leukoencephalopahty [160]; ICH, intracerebral haemorrhage; MELAS, mitochondrial encephalomyopathy, lactic acidosis, and stroke-like episodes; NICD, Notch intracellular domain; RVCL, retinal vasculopathy with cerebral leukodystrophy.

⁎ Several other gene variants of different stroke types and stroke-free white matter hyperintensities have been identified but not widely confirmed by comphrensive meta-analysis approaches or genome-wide association studies. Data summarised from several references [78], [152], [153], [154], [155], [156], [157], [158]. Some of these include genes involved in control of plasma homocysteine and the blood coagulation system.

† Hereditary forms of cerebral small vessel disease (SVD) lead to cognitive impairment or dementia. To date there do not appear to be variants of genes associated with other rarer autosomal dominant or recessive or X-linked monogenic disorders e.g. RVCL, Fabry's, MELAS.

†† SNPs, single nucleotide polymorphisms associated with SVD phenotypes [76], [77].
